# Targeting cytosolic proliferating cell nuclear antigen in neutrophil-dominated inflammation

**DOI:** 10.3389/fimmu.2012.00311

**Published:** 2012-10-09

**Authors:** Alessia De Chiara, Magali Pederzoli-Ribeil, Pierre-Régis Burgel, Claire Danel, Véronique Witko-Sarsat

**Affiliations:** ^1^Department of Immunology and Hematology, INSERM U1016, Cochin Institute ParisFrance; ^2^Paris Descartes UniversityParis, France; ^3^CNRS-UMR 8104Paris, France; ^4^Department of Pneumology, Cochin HospitalParis, France; ^5^Paris Diderot UniversityParis, France; ^6^Department of Pneumology, Bichat HospitalParis, France

**Keywords:** inflammation, neutrophil, apoptosis, PCNA, cystic fibrosis

## Abstract

New therapeutic approaches that can accelerate neutrophil apoptosis under inflammatory conditions to enhance the resolution of inflammation are now under study. Neutrophils are deprived of proliferative capacity and have a tightly controlled lifespan to avoid their persistence at the site of injury. We have recently described that the proliferating cell nuclear antigen (PCNA), a nuclear factor involved in DNA replication and repair of proliferating cells is a key regulator of neutrophil survival. The nuclear-to-cytoplasmic relocalization occurred during granulocytic differentiation and is dependent on a nuclear export sequence thus strongly suggesting that PCNA has physiologic cytoplasmic functions. In this review, we will try to put into perspective the physiologic relevance of PCNA in neutrophils. We will discuss key issues such as molecular structure, post-translational modifications, based on our knowledge of nuclear PCNA, assuming that similar principles governing its function are conserved between nuclear and cytosolic PCNA. The example of cystic fibrosis that features one of the most intense neutrophil-dominated pulmonary inflammation will be discussed. We believe that through an intimate comprehension of the cytosolic PCNA scaffold based on nuclear PCNA knowledge, novel pathways regulating neutrophil survival can be unraveled and innovative agents can be developed to dampen inflammation where it proves detrimental.

## INTRODUCTION

In the acute phase of inflammatory diseases, neutrophils are rapidly recruited to sites of injury or infection where they engulf and kill invading microorganisms (Witko-Sarsat et al., 2000). Moreover, recent studies have underscored an unsuspected neutrophil plasticity that can influence and shape the immune response (Mantovani et al., 2011). Neutrophil apoptosis, the process of programmed cell death that prevents the release of neutrophil histotoxic contents, should be tightly regulated (Kennedy and DeLeo, 2009; Fox et al., 2010) to limit the destructive capacity of neutrophil products to surrounding tissues (Nathan, 2006). The subsequent recognition and phagocytosis of apoptotic neutrophils by macrophages is central to the successful resolution of an inflammatory response. However, it has been reported that neutrophils can phagocytosed apoptotic cells and might participate in the clearance of apoptotic neutrophils at the site of inflammation (Esmann et al., 2010). It is increasingly apparent that the dying neutrophil itself exerts anti-inflammatory effects through modulation of surrounding cell responses (Kobayashi et al., 2002), particularly macrophage inflammatory cytokine release (Ariel and Serhan, 2012). In several inflammatory diseases including arthritis (Wright et al., 2010), vasculitis (Abdgawad et al., 2012), or cystic fibrosis (CF; McKeon et al., 2008; Moriceau et al., 2009,^2010^), neutrophil apoptosis was delayed, thus potentiating the deleterious inflammatory response. Recent studies have highlighted the complexity of neutrophil death mechanisms and uncovered the involvement of novel pathways (Geering and Simon, 2011). Neutrophil survival induced for instance by cytokine such as G-CSF involves a complex gene pattern as evidence by gene array studies (Drewniak et al., 2009). As an example, we have identified the proliferating cell nuclear antigen (PCNA) as a key element controlling neutrophil survival. In neutrophils, that are non-proliferating cells, PCNA localization was strictly cytosolic and correlated with the grade of their viability (Witko-Sarsat et al., 2010).

## A SOPHISTICATED REGULATION OF APOPTOSIS IS REQUIRED TO CONTROL NEUTROPHIL ACTIVATION

Like other cells, a neutrophil possesses both pro-survival and death pathways, the balance of which determines its fate (Witko-Sarsat et al., 2011). Several studies have unraveled the standard cascade of events, which classically include mitochondrial outer membrane permeabilization (MOMP) followed by release of cytochrome c (that is very weak in neutrophils) and other pro-apoptotic proteins into the cytosol, caspase activation, DNA fragmentation, chromatin condensation, loss of membrane asymmetry, formation of apoptotic bodies (Geering and Simon, 2011; Kepp et al., 2011) and, finally, generation of “eat me signals” that stimulate the uptake of apoptotic cells by phagocytes (Paidassi et al., 2009). In neutrophils, the apoptotic machinery presents specific features that render these cells peculiar and very interesting to study as a model in which apoptosis control is cell cycle-independent because they cannot proliferate (Witko-Sarsat et al., 2011). Hence, a complete cell cycle arrest was observed in band cells and segmented neutrophils from bone marrow and in circulating mature neutrophils (Theilgaard-Monch et al., 2005). Expression patterns of apoptosis genes studied by microarray indicated that death control occurred by the p53 pathway in promyelocytes and by death receptor pathways in bone marrow neutrophils. Neutrophil apoptosis is inhibited by a continuous expression of the short lifespan Bcl-2 homolog myeloid cell leukemia-1 (Mcl-1; Thomas et al., 2010). It has been clearly shown that neutrophil survival was regulated by the inducible expression of the short-lived Mcl-1 (Moulding et al., 2001). In that respect, Mcl-1 can be considered as a potential target to modulate neutrophil’s fate (Milot and Filep, 2011). However, the molecular mechanisms controlling this “spontaneous or constitutive” apoptosis still remain obscure: it is not clear whether neutrophil apoptosis occurred because of the lack of external surviving signals or because of its “internal clock”. It has been reported that deactivation of phosphatidylinositol 3,4,5-triphosphate/Akt signaling mediates neutrophil spontaneous death (Zhu et al., 2006). Accordingly, neutrophils depleted of Phosphatase and tensin homolog deleted on chromosome 10 (PTEN), a phosphatase that negatively regulates Akt activity, live much longer than wild-type neutrophils. Some surprising insights into neutrophil survival mechanisms came when cyclin-dependent kinases (CDK) happened to play a key role in the regulation of neutrophil survival (Rossi et al., 2006). Notably, CDK are implicated in the regulation of the cell cycle and constitute targets for anti-cancer therapies (Malumbres et al., 2009). Inhibition of CDK by roscovitine can trigger neutrophil apoptosis by interfering with the phosphorylation of RNA polymerase II and with neutrophil transcrip- tional capacities thereby inducing neutrophil apoptosis (Leitch et al., 2012).

## PCNA: A NOVEL PLAY FOR THIS FASCINATING ACTOR THAT ESCAPES FROM NUCLEUS TO MEDIATE NEUTROPHIL SURVIVAL

An unanticipated finding came with our discovery that PCNA, an ancestral nuclear protein involved in DNA replication, was present in resting neutrophil cytosol and was degraded upon apoptosis. In fact, PCNA happened to be an actor of neutrophil survival (Witko-Sarsat et al., 2010) but it remains to be investigated whether it could regulate “the neutrophil internal clock”. Historically, PCNA was described as an antigen for autoimmune disease in systemic lupus erythematosus patients, detected only in the proliferating cells (Mahler et al., 2012). The tight association of PCNA with cancer transformation resulted in the use of PCNA as a diagnostic and prognostic cell cycle marker in tumors (Stoimenov and Helleday, 2009).

With the aim to start understanding the molecular mechanisms whereby PCNA exerts its anti-apoptotic activities and how the cytosolic PCNA scaffold is regulated, we will next discuss key issues based on our knowledge of nuclear PCNA, assuming that similar principles governing its function are conserved between nuclear and cytosolic PCNA.

### A UNIQUE BUT CONSERVED TRIDIMENSIONAL STRUCTURE

**FIGURE 1:**
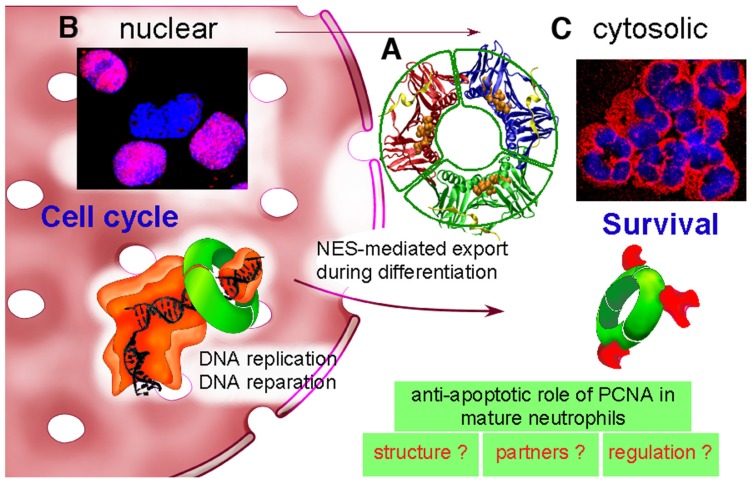
**Dual life of PCNA: from nucleus to cytosol. (A)** PCNA is a scaffold protein, which acts as a trimer (represented here in red–blue–green) as shown by its molecular tridimensional structure (PDB ID: 1VYM, PyMOL Molecular Graphics System). PCNA forms a ring-shaped complex, which can encircle the DNA and orchestrate DNA replication and reparation. PCNA monomers have two similar globular domains, linked by a long flexible loop called the interdomain-connecting loop that binds numerous PCNA partners including p21/waf1. A synthetic peptide from the sequence of p21/waf1 (represented here in yellow) is bound to this interdomain-connecting loop. The nuclear export sequence (NES) is composed of a central helix (represented here with orange balls) localized in the inner face of the PCNA trimer. **(B)** In proliferating cells such as the myeloid CD34^+^ precursor cells, PCNA is nuclear as shown by PCNA immunolabeling (in red) and DNA staining with Hoechst (in blue). The purple color results from the colocalization between PCNA and DNA within nucleus. PCNA is a trimeric ring (represented in green), which exerts its activity during DNA replication by braceleting DNA. PCNA has an important role in DNA synthesis because it is part of the polymerase Pol δ holoenzyme (represented in orange). **(C)** In contrast, in neutrophils, PCNA is localized exclusively within the cytosol as shown by the red color of the cytoplasm and the blue nuclei with its typical polylobular form. During granulocytic differentiation, there is a nuclear-to-cytosol relocalization that involves a NES that we have recently characterized (Bouayad et al., 2012). In neutrophils, under pro-survival conditions, cytosolic PCNA is associated with multiple partners, including procaspases-3, -8, -9, and -10 to prevent their activation, and is stabilized by the treatment of neutrophils with G-CSF (Witko-Sarsat et al., 2010). On the opposite, death signals, either directly or indirectly can target PCNA, leading to a disruption of the PCNA scaffold and thus triggering apoptosis (Witko-Sarsat et al., 2011). Further studies are required to characterize PCNA structure, to identify its partners and to understand how cytosolic PCNA can regulate neutrophil survival.

Proliferating cell nuclear antigen is a ubiquitous protein that has a unique ring-shaped structure (Krishna et al., 1994) and a highly conserved amino acid sequence (Prosperi, 2006). PCNA has been identified in all eukaryotes from unicellular organisms to humans. Striking is the similarity in molecular structure between yeast and human that share 35% amino acid sequences identity but have highly superimposable three-dimensional structure (Stoimenov and Helleday, 2009). Trimeric PCNA exhibits sixfold symmetry as a result of having two globular domains in each monomer (**Figure 1**). The importance of PCNA in DNA replication is tightly linked with its ring-shaped structure that allows to slide freely on duplex DNA (Kelman, 1997). Remarkably, PCNA mutants that cannot form trimers failed to stimulate the polymerase Pol δ (Jonsson et al., 1995). Deletion of the PCNA gene in the yeast showed that PCNA was an essential protein required for DNA replication and knocking out the PCNA gene in mice was lethal (Kelman, 1997).

### PCNA: THE SECOND TO NONE IN THE COORDINATION OF COMPLEX BIOLOGICAL PROCESSES

One key issue is that PCNA has no known enzymatic activity but can interact with a diverse array of proteins and cellular factors to regulate their activities: PCNA has been named the “professional recruiting agent” or more elegantly the “dancer with multiple partners” (Maga and Hubscher, 2003). PCNA-interacting proteins can be classified into two groups (Moldovan et al., 2007): the first consists of enzymes involved in nucleic acid metabolism, including DNA replication [replication factor C (RFC), DNA polymerase δ and ε, FEN-1, DNA ligase I] and repair (MutL homolog 1, MutS homolog 2, Uracil-DNA glycosylase 2) that are localized for the majority exclusively in nucleus. In contrast, the second group consists of cell cycle regulatory proteins (p21/waf1/Cip1, p57, CDK2, growth arrest and DNA damage (Gadd45), and the myeloid-differentiation primary-response (MyD118), Mcl-1) that localized both within the nuclei or the cytoplasmic compartment depending on the cell type. Except for Mcl-1, the major anti-apoptotic Bcl-2 homolog expressed in neutrophils (Thomas et al., 2010), these latter PCNA partners have not been studied in the neutrophil survival context. We have previously shown that, in mature neutrophils, PCNA was constitutively associated with procaspase-3, procaspase-8, procaspase-9, and procaspase-10, presumably sequestering them within the cytosol to prevent their activation. In line with this notion, recombinant PCNA was shown to interfere with in vitro procaspase 9 activation (Witko-Sarsat et al., 2010), thus strongly suggesting that PCNA association with procaspases represents a way to block their activation.

### p21/waf1 DESTABILIZED THE PCNA SCAFFOLD AND TRIGGERED NEUTROPHIL APOPTOSIS

p21/waf1 is a well characterized PCNA partner that has been identified in a protein complex containing PCNA, cyclin, and CDK (Xiong et al., 1992; Waga et al., 1994). p21/waf1 is a p53-responsive gene but p21 expression can also be p53-independent (Biggs and Kraft, 1995). The p21 has two different inhibitory effects on the entry of the cell into S-phase. One is to inhibit the kinase activity of CDK and the other is to inhibit DNA replication via an interaction with PCNA (Goubin and Ducommun, 1995). Based on previous studies, synthetic peptides such as the carboxy-p21 peptide (residues 141–160 on the p21/waf1 sequence) carrying the consensus sequence for binding to the PCNA interdomain-connecting loop (**Figure 1**) was shown to act as an effective competitor for PCNA partners and to interfere with its functions (Warbrick, 2000). Remarkably, this carboxy-p21 triggered neutrophil apoptosis and concomitant PCNA degradation, in addition to impair the capacity of G-CSF to prolong neutrophil survival in vitro (Witko-Sarsat et al., 2010). Thus, the observation that the carboxy-p21 triggered neutrophil apoptosis by disturbing the PCNA scaffold clearly showed to us that PCNA is pivotal in maintaining neutrophil survival. Whether p21/waf1 expression controls the PCNA scaffold in neutrophils has not been investigated yet. The expression of p21 has been shown to be downregulated during granulocytic differentiation (Yaroslavskiy et al., 1999) and its expression in mature neutrophils is low under resting conditions (Klausen et al., 2004). Surprisingly, p21 mRNA has been shown to be strongly upregulated *in vivo* in human neutrophils isolated from bronchoalveolar lavages following LPS intratracheal instillation (Coldren et al., 2006). Whether p21–PCNA interaction could play a role in neutrophil survival will require further investigations.

### CYTOSOL AS A PHYSIOLOGIC PLAYGROUND FOR PCNA ACTION

The peculiar exclusive cytoplasmic localization was a feature of mature neutrophils as, for instance, PCNA was detectable exclusively in the nucleus of CD34^+^ cells or in myeloblasts isolated from human bone marrow aspirates (Witko-Sarsat et al., 2010). More recently we have provided evidence of an active PCNA nuclear export that involved the chromosome region maintenance 1 (CRM1) exportin (Turner et al., 2012). Accordingly, leptomycin B, an inhibitor of the CRM1 exportin inhibited this PCNA relocalization during granulocytic differentiation of human primary CD34^+^ cells or in promyelocytic cell lines (Bouayad et al., 2012). Using enhanced green fluorescent protein fusion constructs, we demonstrated that PCNA relocalization involved a nuclear export signal (NES) located from I11 to I23 in the PCNA sequence. However, this NES, located at the inner face of the PCNA trimer (**Figure 1**) was not functional in wild-type PCNA, but instead, was fully active and leptomycin B-sensitive in the monomeric PCNAY114A mutant. We also provided evidence that nuclear-to-cytoplasmic relocalization that occurred physiologically during myeloid differentiation was essential for PCNA anti-apoptotic activity in mature neutrophils. It is noteworthy that the PCNA NES was extremely conserved between species (Bouayad et al., 2012) thus suggesting that this CRM1-dependent export of PCNA was part of the physiologic PCNA trafficking presumably occurring in cells other than neutrophils, thus uncovering a novel aspect of PCNA functions. Notably, the presence of PCNA has been recently described in the cytosol of cancer cells (Naryzhny and Lee, 2010).

### POST-TRANSLATIONAL MODIFICATIONS OF CYTOSOLIC PCNA: A KEY IN NEUTROPHIL SURVIVAL?

Another level of complexity in the ballet of PCNA partners within nucleus, is the multiplicity of PCNA post-translational modifications that modulate specific protein interactions (Moldovan et al., 2007). In fact, phosphorylation (although controversial), ubiquitination, sumoylation, and acetylation that have been described for nuclear PCNA offer a great deal of options to modulate PCNA activities (Naryzhny and Lee, 2004). In neutrophils, we have observed that PCNA was ubiquitinated and was degraded via the proteasome during apoptosis (Witko-Sarsat et al., 2010). The levels of PCNA were found to time-dependently decrease in neutrophils undergoing apoptosis regardless of whether the triggering signaling cascade passed through the extrinsic (death receptors) or the intrinsic pathway (mitochondria). Since proteasome inhibitors reversed such a PCNA diminution, we concluded that a proteasome-mediated PCNA degradation, triggered along both the death receptor and mitochondrial apoptotic cascades, was responsible for apoptosis-induced PCNA degradation.

## MODULATING PCNA SCAFFOLD IN NEUTROPHIL-DRIVEN INFLAMMATION: THE MODEL OF CYSTIC FIBROSIS

### THE PROMINENT ROLE OF NEUTROPHIL IN CYSTIC FIBROSIS AIRWAY INFLAMMATION

Cystic fibrosis which is a lethal autosomal recessive disorder caused by mutation of the CF transmembrane regulator (CFTR) gene, is characterized by an intense neutrophil-dominated airway inflammation (Cantin, 1995) and a chronic bacterial colonization with *Pseudomonas aeruginosa*. Plugging in small airways contributes to the morbidity and mortality in CF (Burgel et al., 2007), leading to respiratory failure and the need for lung transplantation (Burgel and Nadel, 2008). The prognosis is tightly linked with the severity of the inflammatory process. Hence, the extraordinary numbers of neutrophils accumulating within airways of CF patients has led to the hypothesis of an innate immunity failure (Bals et al., 1999). Today, the current treatment involves antibiotherapy and mucolytic drugs but therapeutic intervention in CF remains a challenge (Pier, 2012). Anti-inflammatory drugs for CF lung disease appear to have some beneficial effects on disease progression. These agents include oral corticosteroids and ibuprofen, as well as azithromycin, which, in addition to its antimicrobial effects, also possess anti-inflammatory properties. Inhaled corticosteroids, antioxidants, nutritional supplements, and protease inhibitors have a limited impact on the disease. Adverse effects limit therapy with oral corticosteroids and ibuprofen (Narasimhan and Cohen, 2011). Hence, the lack of promising candidate emphasizes the need for fresh approaches in the management of airway inflammation in CF, for instance by targeting neutrophil apoptosis in combination with antibiotherapies.

Previous studies on CF patient’s neutrophils indicated functional disturbances in bacterial phagocytosis, killing, and other effector functions (Downey et al., 2009). Because of the extreme heterogeneity of CF patients in terms of infectious status (Witko-Sarsat et al., 1999), the comparisons of experiments and results are difficult. Indeed, we have previously shown that neutrophils from CF parents who were heterozygous for CFTR mutation, had also disturbed neutrophil functions thus suggesting the possibility of an innate neutrophil defect in CF (Witko-Sarsat et al., 1996; Moriceau et al., 2010). Accordingly, a recent study has provided evidence that the absence of the CFTR from myeloid-derived cells slows the resolution of inflammation (Bonfield et al., 2012). Gene-expression patterns of neutrophils from clinically stable and healthy controls have shown dramatic differences (Adib-Conquy et al., 2008). This was consistent with a perturbed “inflammatory program” in CF neutrophils (Hayes et al., 2011), which remains to be investigated (Tirouvanziam et al., 2008). It should also be mentioned that perpetuation of inflammation in the CF airway may also be amplified by defective macrophage clearance mechanisms. It has been shown that persistence of infection in CF was partly due to ineffective uptake and killing of pathogens due to a defective macrophage innate response (Wright et al., 2009). Notably, a defect in apoptotic cell clearance has also been reported in CF (Vandivier et al., 2002).

### DYSREGULATED NEUTROPHIL APOPTOSIS: A POTENTIAL TARGET FOR THERAPEUTIC INTERVENTION

**FIGURE 2:**
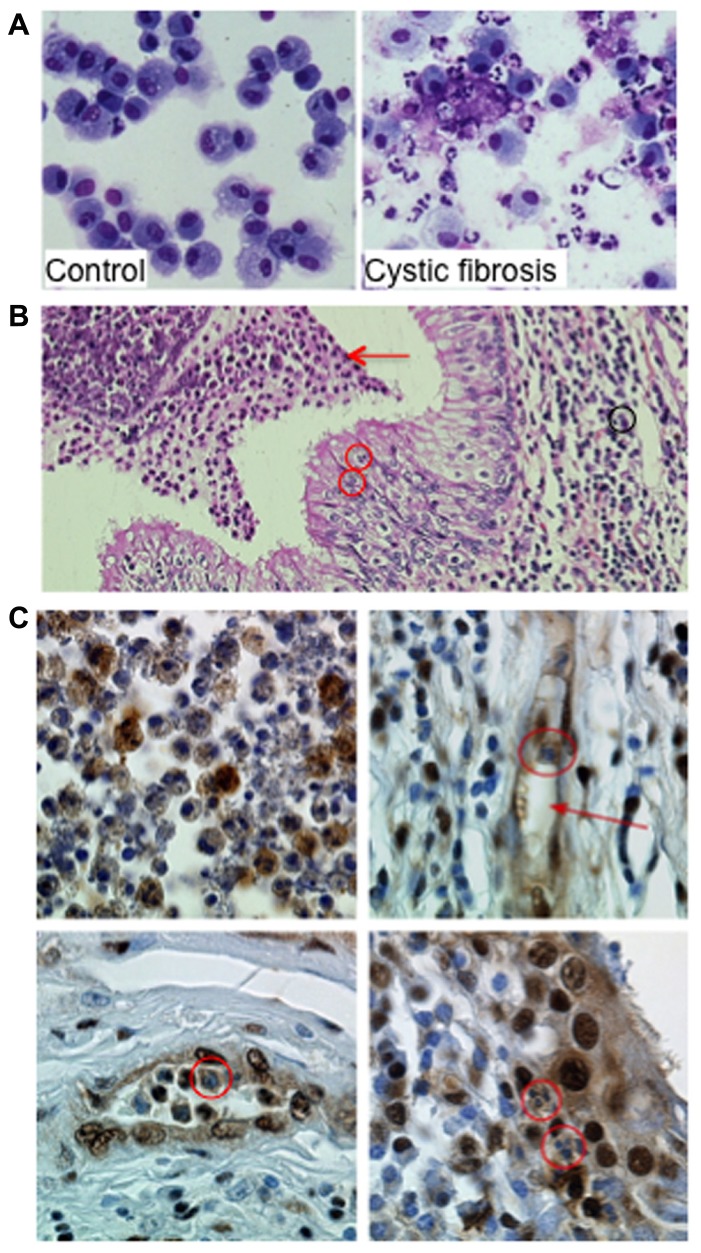
**Proliferating cell nuclear antigen (PCNA) expression in neutrophils within airways from CF patients. (A)** Neutrophils and macrophages from bronchoalveolar lavages (BAL) from a control donor and from a patient with CF. The control BAL was composed mainly of alveolar macrophages. In contrast, in BAL from CF 95% of the cells are neutrophils either viable or apoptotic. Few macrophages and lymphocytes are present as well as cell debris and mucus (Giemsa stain ×630 original magnification). **(B)** Neutrophil-dominated inflammation in lung explants from a CF patient. Acute inflammation within the lung in CF patient with characteristic histologic features: (1) inflammatory infiltrate with neutrophils (black circle and arrow) and lymphocytes in the lamina propria, (2) transepithelial neutrophil migration (red circles), and (3) accumulation of degenerating and apoptotic neutrophils in the lumen (red arrow; Hematoxylin–Eosin–Safran staining ×200 original magnification). Lung explant specimens from CF patients were obtained at transplantation (Hôpital Européen Georges Pompidou). **(C)** Immunoperoxidase labeling of PCNA on paraffin sections of lung explants from a CF patient. Labeling was performed using a rabbit polyclonal anti-PCNA antibody (Ab5, diluted 1:100, Calbiochem) and immunoperoxidase detection (Dako) as previously described (Moriceau et al., 2009; ×630 original magnification). The airway lumen contents neutrophils expressing PCNA (upper left panel). Neutrophils within vessels (red arrow, upper right panel, red circle on the lower left panel) expressed high amounts of PCNA as did neutrophils found in the epithelium (red circle on lower right panel). This strong PCNA expression in neutrophil cytosol contrasts with the lack of labeling observed in cells present in the lamina propria, including fibroblasts and lymphocytes. Similar observations were made in lung explants from four CF patients.

Given the number of neutrophils in CF airways (Danel et al., 1996; **Figure 2A**), late neutrophil apoptosis could have devastating consequences. This neutrophil-dominated airway inflammation typical of the CF condition is representative of a chronic but active inflammatory process with neutrophil persistence suggesting a defect in apoptotic neutrophil clearance by macrophages (**Figure 2B**). Indeed, we (Moriceau et al., 2010) and others (McKeon et al., 2008) reported that neutrophils from CF patients undergo delayed apoptosis and have decreased levels of the pro-apoptotic protein Bax (Dibbert et al., 1999), thereby slowing their removal by macrophages and potentiating airway inflammation. In an attempt to modulate the delayed apoptosis in neutrophils from CF patients, roscovitine was used at 10 μM to restore normal apoptosis levels for CF PMN (Moriceau et al., 2010). Whether CDK-mediated survival pathway could crosstalk with the PCNA scaffold is currently unknown and would require more investigations.

We recently identified coronin-1A (Grogan et al., 1997) as a cytosolic protein overexpressed in CF neutrophils, a finding that was consistent with its anti-apoptotic function (Moriceau et al., 2009). Coronin-1 expression investigated by immunohistochemistry of pulmonary tissues obtained from CF patients during transplantation clearly showed a strong coronin-1A expression in neutrophils at the site of inflammation (Moriceau et al., 2009). Similar immunohistochemistry labeling of PCNA in neutrophils within the airway lumen showed a great variation in their PCNA contents reflecting their different apoptosis rates (**Figure 2C**). In contrast, the cytoplasm of neutrophils present within the mucosa, lamina propria and vessels were strongly labeled thus indicating their survival state (**Figure 2C**). These observations suggest that PCNA was highly expressed in neutrophils infiltrating the lung of CF patients and might play a role in neutrophil survival at the site of inflammation. Whether PCNA could be associated to coronin-1A is currently unknown but should require more investigation. Investigation of cytosolic PCNA within neutrophils at sites of inflammation (for instance in vasculitis or rheumatoid arthritis) should also be explored.

Strategies aiming at potentiating neutrophil apoptosis by targeting the PCNA scaffold in CF have to be carefully investigated and could be combined with other anti-inflammatory or anti-infectious therapeutic strategies to achieve a maximum efficacy in term of dampening neutrophil-driven inflammation. It might be possible to adjust this type of therapy to avoid any risk of neutropenia-induced infection.

## CONCLUSION

Highlighting peculiar pathways used by neutrophils to control their survival (Geering and Simon, 2011) are of pivotal importance for the development of novel anti-inflammatory strategies (Duffin et al., 2010). Even in the absence of proliferation, PCNA seems to have a conservative function for preserving neutrophil’s life. This particular cytosolic PCNA localization strongly suggests that this could be harnessed for therapeutic purposes when neutrophils would be out of control such as in sustained inflammation. This will be our main challenge to exploit all the data on nuclear PCNA gathered during more than five decades of work, and try to be creative to understand how the enigmatic PCNA scaffold participates to neutrophil survival.

## Conflict of Interest Statement

The authors declare that the research was conducted in the absence of any commercial or financial relationships that could be construed as a potential conflict of interest.
